# Gestational malaria associated to *Plasmodium vivax *and *Plasmodium falciparum *placental mixed-infection followed by foetal loss: a case report from an unstable transmission area in Brazil

**DOI:** 10.1186/1475-2875-10-178

**Published:** 2011-06-27

**Authors:** Bruna O Carvalho, Joycenéa S Matsuda, Sergio LB Luz, Flor E Martinez-Espinosa, Juliana A Leite, Fernanda Franzin, Patrícia P Orlandi, Gustavo B Gregoracci, Marcus VG Lacerda, Paulo A Nogueira, Fabio TM Costa

**Affiliations:** 1 Departamento de Genética, Evolução e Bioagentes, Universidade Estadual de Campinas (UNICAMP) - Campinas, SP, Brazil; 2 Instituto Oswaldo Cruz, Fundação Oswaldo Cruz (FIOCRUZ) - Manaus, AM, Brazil; 3 Fundação de Medicina Tropical do Amazonas (FMT-AM) - Manaus, AM, Brazil; 4 Universidade do Estado do Amazonas (UEA) - Manaus, AM, Brazil; 5 Centro Universitário Nilton Lins - Manaus, AM, Brazil

## Abstract

Gestational malaria is a multi-factorial syndrome leading to poor outcomes for both the mother and foetus. Although an unusual increasing in the number of hospitalizations caused by *Plasmodium vivax *has been reported in Brazil, mortality is rarely observed. This is a report of a gestational malaria case that occurred in the city of Manaus (Amazonas State, Brazil) and resulted in foetal loss. The patient presented placental mixed-infection by *Plasmodium vivax *and *Plasmodium falciparum *after diagnosis by nested-PCR, however microscopic analysis failed to detect *P. falciparum *in the peripheral blood. Furthermore, as the patient did not receive proper treatment for *P. falciparum *and hospitalization occurred soon after drug treatment, it seems that *P. falciparum *pathology was modulated by the concurrent presence of *P. vivax*. Collectively, this case confirms the tropism towards the placenta by both of these species of parasites, reinforces the notion that co-existence of distinct malaria parasites interferes on diseases' outcomes, and opens discussions regarding diagnostic methods, malaria treatment during pregnancy and prenatal care for women living in unstable transmission areas of malaria, such as the Brazilian Amazon.

## Background

In severe cases of *Plasmodium falciparum *infection, clinical complications are associated with the sequestration of *P. falciparum*-infected erythrocytes (Pf-iE) within microvasculature and placental syncytiotrophoblasts [1-5]. Vivax malaria has long been considered a benign infection; however, the malaria pigment of this species has been detected in the placenta of *Plasmodium vivax*-infected women [6]. Further, pregnant women infected with *P. vivax *experience maternal anaemia, and some of their babies present a low birth weight [6,7], which are clinical features frequently associated with Pf-iE placental adhesion [1,2]. Despite the adverse pregnancy outcomes associated with *P. vivax *infection [6], information concerning epidemiology and clinical consequences of vivax malaria during pregnancy is lacking.

In Brazil, where malaria incidence is almost exclusively restricted to the Amazon (99.8% of the cases), *P. vivax *was responsible for the majority (83.7%) of registered cases in 2008. *Plasmodium falciparum *infections accounted for 16.3% of cases, and *Plasmodium malariae *infection was rarely observed [8]. Additionally, chloroquine-resistant strains of *P. vivax *have emerged in the Brazilian Amazon [9].

## Case presentation

A 19-year-old pregnant woman, estimated to be 35 weeks of gestation (WG), living on the boundary of the city of Manaus - Amazon State (3.09S, 59.58W), surrounded by the Amazon rainforest, was diagnosed for *P. vivax *infection at the nearest Health Center and showed approximately 90,000 parasites/mm^3^. In Brazil, the microscopic examination of Giemsa-stained thick blood smear is the official method for malaria diagnosis. This was her fourth pregnancy, and she had no medical history of previous abortion, stillbirth or pre-term delivery. The patient had three previous malaria episodes, the last occurring two years ago. Additionally, she reported a plasmodial infection during her third pregnancy. At the time, the patient was treated and no further complications were observed.

She was given a three-day regimen (25 mg/kg) of oral chloroquine, with four pills (150 mg each) administered in the first day, followed by three pills on the two subsequent days. However, after the second dose, the patient presented with vomiting, which led to cessation of the drug treatment. The patient was subsequently transferred to a tertiary-care maternity hospital in Manaus, where she was hospitalized until delivery. At the maternity hospital, the patient presented symptoms of fever, headache, jaundice, anorexia, chills and hypertension. Urine sediment analysis revealed that bilirubin and biliary pigments were three-fold above the standard levels. Furthermore, blood analysis revealed slight anaemia (Ht 29.3%, Hb 10.1 g/dL) and leukocyte count were normal (4,200 cells/mm^3^), with 67% lymphocytes. Serological tests for syphilis, toxoplasmosis, measles and HIV were negative.

Two days after patient admission, another thick blood smear was performed and no patent parasitaemia was observed. Ultrasound analysis showed that foetal heart rate tracings were stable and normal. While foetal centralization was not observed, the ultrasound did reveal oligohydramnios (amniotic fluid index < 5.0 cm), abnormal foetal symmetry and abnormal placental texture. Although pregnancy was estimated to be 35 WG, with a foetal weight of 2,500 g, according to the patient's last menstruation date, foetal growth was approximately 38-39 WG. Thus, the estimate of 35 WG after ultrasound analysis might represent impairment of intra-uterine growth. Two days later in a routine follow-up, an abnormal foetal heart rate was observed. Another ultrasound analysis was performed, and no foetal heartbeat was detected, and oligohydramnios was observed. Next, labour was induced by administration of oxytocin, and foetal loss of a male weighing 2,670 g was confirmed. No foetal autopsy was performed due to the lack of authorization by relatives. Macroscopic examination of the placenta revealed an abnormal dark colour; following patient consent, a sample of the placental tissue was collected for further microscopic and molecular analysis. Molecular analysis of the placenta revealed a mixed infection with *P. falciparum *and *P. vivax*. Taken together, these findings suggest placental dysfunction most likely associated with plasmodial infection, as other common infectious diseases that cause the same phenomenon were ruled out.

**Figure 1:**
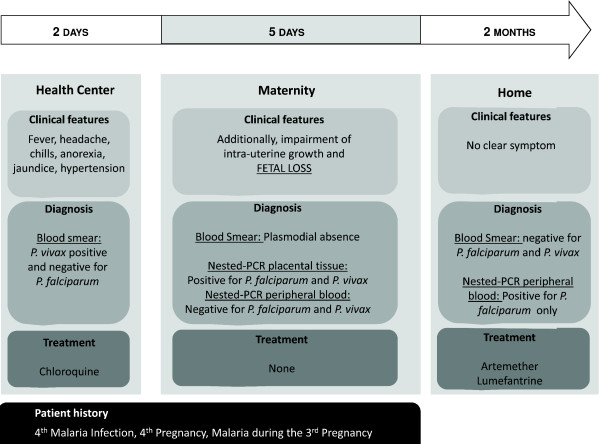
**Schematic representation of the major events reported in this case**. Diagnosis, treatment and symptoms are reported as they occurred in a chronological manner. Following the *P. vivax *positive blood smear diagnosis, the patient remained hospitalized until delivery, when placental tissue and mother's peripheral blood samples were collected and molecular analyses were performed. Two months after foetal loss, a new blood sample was collected, allowing *P. falciparum *molecular diagnosis by nested-PCR.

Because of the absence of parasite forms in the thick blood smear performed at the maternity, the patient did not receive any anti-malarial treatment during her stay and immediately after being released from the hospital. In the second month after foetus loss, nested-PCR analysis was conducted on the asymptomatic mother's peripheral blood and a *P. falciparum*-specific PCR product was detected, despite the maintenance of negative thick blood smears. At the time, the patient was treated with artemether/lumefantrine for three days. Figure 1 summarizes, in a chronological manner, the major events reported in this case.

## Methods

### Molecular analysis

A small fraction (1 × 1 × 1 cm) of maternal placenta was collected, frozen in liquid nitrogen and crushed in 2 mL of digestion buffer (100 mM NaCl, 10 mM Tris-HCl, 25 mM EDTA and 0.5% SDS) before incubation overnight at 37°C in the presence of proteinase K (0.1 mg/mL, Sigma). The DNA used for PCR amplification was purified by two phenol/chloroform extractions, followed by ethanol precipitation; DNA samples were resuspended in water. Two samples of the patient's peripheral blood were collected, the first one at few hours after the foetus loss and other approximated two months later. Genomic DNA (gDNA) was purified using the Charge Switch gDNA Blood Kit (Invitrogen) according to the manufacturer's protocol. To determine and discriminate the presence of *Plasmodium *spp. gDNA in the blood sample, sensitive nested-PCR was performed using species-specific oligonucleotides based on human malaria parasite genes for the 18S small subunit ribosomal RNA (ssrRNA) as described previously [10]. Important, this set of oligonucleotides amplifies small amounts of genetic material only from viable parasites [10]. As a negative control, gDNA purified from a healthy placenta or from the peripheral blood of a non-infected individual was used. The products of these reactions were analyzed on a 2% agarose gel stained with ethidium bromide. To ensure nested-PCR specificity, amplification products were directly cloned into the pGEM-T cloning vector kit (Promega) and then analysed with the aid of the MegaBace 500 automatic sequencer (GE - Health care).

Nested-PCR results conducted with patient's placental tissue were also confirmed by semi-nested multiplex malaria PCR (SnM-PCR) (Additional file 1) assays as described [11]. Because of the poor conditions of the placental tissue collected histological analyses were not conducted.

## Consent

Written and informed consent was sought and granted from the patient who attended the tertiary-care maternity hospital.

## Results

**Figure 2:**
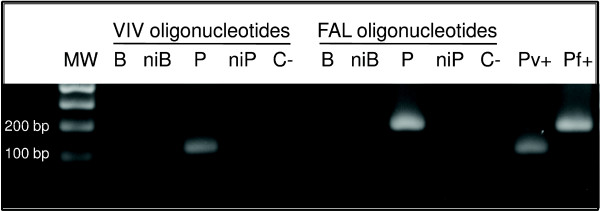
**The presence of *P. falciparum- *and *P. vivax*-infected erythrocytes in the placenta collected after foetal loss**. Agarose gel electrophoresis of nested-PCR amplified products in the presence of species-specific human malaria parasite oligonucleotides (VIV or FAL, specific for *P. vivax *or *P. falciparum*, respectively) based on the parasite small subunit ribosomal RNA (ssrRNA) gene. Both *P. vivax *and *P. falciparum *gDNA were found in the placenta; no plasmodial gDNA was detected in peripheral blood after delivery. Abbreviations are as follows: **MW**, molecular weight; **bp**, base pair; **B**, patient peripheral blood sample; **niB**, non-infected human peripheral blood; **P**, patient placenta sample; **niP**, non-infected human placenta; **C-**, negative control, absence of nuclear material; **Pv+ **and **Pf+**, positive controls representing amplification product of *P. vivax *(120 bp) and *P. falciparum *(200 bp) ssrRNA gene.

The patient was diagnosed for *P. vivax *infection by thick blood smear performed in a Health Center during her pregnancy. To confirm this diagnosis and to investigate placental vivax infection, we conducted nested-PCR analysis of the placental tissue using species-specific oligonucleotides. As expected, specific amplification of *P. vivax *18S ssrRNA gene was detected (Figure 2). However, a positive reaction was also observed after incubation with *P. falciparum*-specific oligonucleotides (Figure 2); thus revealing a mixed-species placental infection. Despite the positive nested-PCR result, *P. falciparum*-infected erythrocytes were not detected in peripheral blood by the thick blood smear before delivery. Nested-PCR analysis of peripheral blood was also performed immediately after foetal loss, and neither *P. vivax- *nor *P. falciparum*-specific fragments were observed (Figure 2).

## Discussion

Recent efforts by Brazilian authorities have led to a significant reduction in malaria cases (456,000 in 2007 to 314,000 in 2009) and in the Amazonian Annual Parasitological Index (API; 31.9 in 1999 to 12.8 in 2008) over the last few years [8]. Nevertheless, disease incidence in women and children less than 10 years of age increased from 2003 to 2008; disease in women increased from 34.9 to 38.6%, while the incidence in children under 10 increased from 22 to 25.2% [8]. Indeed, in Manaus, an unusual augmentation in the number of hospitalizations of *P. vivax*-infected individuals has been reported over the past years [12]. Brazilian malaria treatment policy restricts the use of antimalarials to confirmed parasitological cases. A combination of chloroquine and primaquine is used in uncomplicated *P. vivax *infections, while artemether-lumefantrine is the choice to treat uncomplicated *P. falciparum *infections. In severe falciparum malaria, intravenous administration of artemisinin derivatives is recommended [13].

The relationship between *P. vivax *infection and pregnancy outcomes such as stillbirth and miscarriage remains unclear in the literature. However, studies conducted in Southeastern Asia indicate a correlation between *P. vivax *infection and adverse pregnancy outcomes, eventually leading to maternal anemia and low birth weight, and with the presence of malaria pigment in the placenta [6,7]. Furthermore, it has been shown an increase in the frequency of *P. falciparum *infections in pregnant women in a cohort of 1,699 childbearing women in the Brazilian Amazon [14].

This is a case report of gestational malaria with foetal loss, in which DNA of both *P. vivax *and *P. falciparum *were amplified from infected placental tissue. In contrast, only *P. vivax*-infected erythrocytes (Pv-iE) were found in patient peripheral blood following diagnosis by Giemsa-stained thick blood smear. Although the patient's chloroquine treatment was incomplete, the utilized diagnostic method failed to detect *P. vivax *in the peripheral blood after treatment; thus showing some efficacy of the drug treatment against this species of parasite. Moreover, as the nested-PCR analysis conducted by us detects only viable parasites, we assume that both of these species of parasites were accumulated or sequestered in the placenta and played a role in pathology.

During the months that followed foetus loss, even without a clear symptom, the patient was further tested for malaria infection by thick blood smear in the health center, and parasites were not detected in the peripheral blood. However, a molecular diagnosis by nested-PCR performed in the patient's peripheral blood collected two months after delivery detected only *P. falciparum *(Additional file 2). Although immunological analyses were not carried out, microscopically undetectable levels of peripheral parasitaemia might suggest that this woman presents an important acquired immunity against *P. falciparum*.

It is not known by which means *P. vivax *can lead to severe complications in pregnancy; even though a recent report has shown the ability of Pv-iE to cytoadhere to placental cryosections [15]. However, several reports have shown that mixed-malaria species infection attenuates the severity of *P. falciparum *pathology in Asia and in Brazil [16-18]. Moreover, it has been recently shown that in malaria mixed-species infections patients develop higher levels of fever and antisera against *P. vivax *and *P. falciparum *in comparison to patients with a single infection [19], and competition between co-infecting parasites for limited resources within a host consists in a form of selective pressure directed from one species to another [20]. Given that the patient was hospitalized soon after chloroquine administration, it is most likely that by disrupting the equilibrium between these two parasites an exacerbation of the clinical signs by the most virulent species contributed to foetal death. Nonetheless, the possibility that *P. falciparum *erythrocytic infection has occurred in the period between chloroquine treatment and delivery cannot be ruled out.

## Conclusions

Collectively, this case reinforces the notion that by interfering in the homeostasis of two distinct species of *Plasmodium *parasites in mixed-infections symptoms provoked by the more virulent species can exacerbate. Moreover, it demonstrates the necessity during pregnancy to consider asymptomatic plasmodial infections as a potential complication, and urges careful patient follow-up even when peripheral parasitaemia seems absent following drug treatment. Highly sensitive techniques are available and must be used as diagnostic tools, especially in high-risk groups such as pregnant women.

## Competing interests

The authors declare that they have no competing interests.

## Authors' contributions

BOC, JSM, PAN were enrolled in collecting the clinical records and in interviewing the maternity and malaria health center staff. BOC, JAL, FF, PPO, GBG and SLBL set up the placental tissue DNA extraction and fragments cloning, performed and interpreted nested-PCR and sequencing data. BOC, MVGL, FEM and PAN conceived the study, contributed in its coordination and helped to draft the manuscript. FTMC contributed to the study design and coordination, helped to interpret the data and to draft the final version of the manuscript. All authors have read and approved the final manuscript.

## Supplementary Material

Additional file 1 **Presence confirmation of *P. falciparum- *and *P. vivax*-infected erythrocytes in the placental tissue collected after foetal loss**. Agarose gel electrophoresis of semi-nested multiplex PCR (SnM-PCR) amplified products in the presence of species-specific human malaria parasite oligonucleotides specific for *P. ovale*, *P. malariae*, *P. vivax *or *P. falciparum *ssrRNA gene. Both *P. vivax *and *P. falciparum *gDNA were found in the placenta. Abbreviations are as follows: **MW**, molecular weight; **bp**, base pair; **S**, patient placental sample; **C-**, negative control, absence of nuclear material; **Po+**, **Pm+**, **Pv+ **and **Pf+**, positive controls representing amplification product of *P. ovale *(436 bp), *P. malariae *(269 bp), *P. vivax *(499 bp) and *P. falciparum *(395 bp) ssrRNA gene. Click here for file

Additional file 2 **Molecular diagnosis of *Plasmodium falciparum *infection performed two months after delivery**. Agarose gel electrophoresis representing nested-PCR performed in the presence of patient's peripheral blood collected two months after delivery and species-specific human malaria parasite oligonucleotides (VIV or FAL, specific for *P. vivax *or *P. falciparum *ssrRNA gene, respectively). Only *P. falciparum *gDNA was detected in peripheral blood. Abbreviations are as follows: **MW**, molecular weight; **bp**, base pair; **B**, patient peripheral blood sample; **niB**, non-infected peripheral blood sample; **C-**, negative control, absence of nuclear material; **Pv+ **and **Pf+**, positive controls representing amplification product of *P. vivax *(120 bp) and *P. falciparum *(200 bp) ssrRNA gene. Click here for file

## References

[B1] Buffet PA Gamain B Scheidig C Baruch D Smith JD Hernandez-Rivas R Pouvelle B Oishi S Fujii N Fusai T Parzy D Miller LH Gysin J Scherf A *Plasmodium falciparum *domain mediating adhesion to chondroitin sulfate A: a receptor for human placental infection Proc Natl Acad Sci USA 1999 96 12743 12748 10.1073/pnas.96.22.12743 10535993 PMC23079

[B2] Scherf A Pouvelle B Buffet PA Gysin J Molecular mechanisms of *Plasmodium falciparum *placental adhesion Cell Microbiol 2001 3 125 131 10.1046/j.1462-5822.2001.00109.x 11260135

[B3] Andrews KT Lanzer M Maternal malaria: *Plasmodium falciparum *sequestration in the placenta Parasitol Res 2002 88 715 723 10.1007/s00436-002-0624-5 12122428

[B4] Kraemer SM Smith JD A family affair: var genes, PfEMP1 binding, and malaria disease Curr Opin Microbiol 2006 9 374 380 10.1016/j.mib.2006.06.006 16814594

[B5] Costa FT Avril M Nogueira PA Gysin J Cytoadhesion of *Plasmodium falciparum*-infected erythrocytes and the infected placenta: a two-way pathway Braz J Med Biol Res 2006 39 1525 1536 10.1590/S0100-879X2006001200003 17160261

[B6] Nosten F McGready R Simpson JA Thwai KL Balkan S Cho T Hkirijaroen L Looareesuwan S White NJ Effects of *Plasmodium vivax *malaria in pregnancy Lancet 1999 354 546 549 10.1016/S0140-6736(98)09247-2 10470698

[B7] Price RN Tjitra E Guerra CA Yeung S White NJ Anstey NM Vivax malaria: neglected and not benign Am J Trop Med Hyg 2007 77 Suppl 6 79 87 18165478 PMC2653940

[B8] Oliveira-Ferreira J Lacerda MV Brasil P Ladislau JL Tauil PL Daniel-Ribeiro CT Malaria in Brazil: an overview Malar J 2010 9 115 10.1186/1475-2875-9-115 20433744 PMC2891813

[B9] de Santana Filho FS Arcanjo AR Chehuan YM Costa MR Martinez-Espinosa FE Vieira JL Barbosa MG Alecrim WD Alecrim MG Chloroquine-resistant *Plasmodium vivax*, Brazilian Amazon Emerg Infect Dis 2007 13 1125 1126 18214203 10.3201/eid1307.061386 PMC2878224

[B10] Snounou G Singh B Nested PCR analysis of *Plasmodium *parasites Meth Mol Med 2002 72 189 203 10.1385/1-59259-271-6:189 12125116

[B11] Rubio JM Post RJ van Leeuwen WM Henry MC Lindergard G Hommel M Alternative polymerase chain reaction method to identify *Plasmodium *species in human blood samples: the semi-nested multiplex malaria PCR (SnM-PCR) Trans R Soc Trop Med Hyg 2002 96 Suppl 1 S199 204 12055839 10.1016/s0035-9203(02)90077-5

[B12] Santos-Ciminera PD Roberts DR Alecrim MG Costa MR Quinnan GV Jr Malaria diagnosis and hospitalization trends, Brazil Emerg Infect Dis 2007 13 1597 1600 18258018 10.3201/eid1310.070052 PMC2851511

[B13] Ministério da Saúde - Brazil http://portal.saude.gov.br/portal/saude

[B14] Martínez-Espinosa FE Daniel-Ribeiro CT Alecrim WD Malaria during pregnancy in a reference centre from the Brazilian Amazon: unexpected increase in the frequency of *Plasmodium falciparum *infections Mem Inst Oswaldo Cruz 2004 99 19 21 10.1590/S0074-02762004000100003 15057341

[B15] Carvalho BO Lopes SC Nogueira PA Orlandi PP Bargieri DY Blanco YC Mamoni R Leite JA Rodrigues MM Soares IS Oliveira TR Wunderlich G Lacerda MV Del Portillo HA Araújo MO Russell B Suwanarusk R Snounou G Rénia L Costa FT On the cytoadhesion of *Plasmodium vivax*-infected erythrocytes J Infect Dis 2010 202 638 647 10.1086/654815 20617923

[B16] Snounou G White NJ The co-existence of Plasmodium: sidelights from falciparum and vivax malaria in Thailand Trends Parasitol 2004 20 333 339 10.1016/j.pt.2004.05.004 15193565

[B17] Luxemburger C Ricci F Nosten F Raimond D Bathet S White NJ The epidemiology of severe malaria in an area of low transmission in Thailand Trans R Soc Trop Med Hyg 1997 91 256 262 10.1016/S0035-9203(97)90066-3 9231189

[B18] Lorenzetti A Fornazari PA Bonini-Domingos AC de Souza Rodrigues Penhalbel R Fugikaha E Bonini-Domingos CR Fraga VD Conceição LM Rossit AR Cavasini CE Couto VS Machado RL Mixed *Plasmodium falciparum *infections and its clinical implications in four areas of the Brazilian Amazon region Acta Trop 2008 107 8 12 10.1016/j.actatropica.2008.03.012 18468567

[B19] Chuangchaiya S Jangpatarapongsa K Chootong P Sirichaisinthop J Sattabongkot J Pattanapanyasat K Chotivanich K Troye-Blomberg M Cui L Udomsangpetch R Immune response to *Plasmodium vivax *has a potential to reduce malaria severity Clin Exp Immunol 2010 160 233 239 20030672 10.1111/j.1365-2249.2009.04075.x PMC2857946

[B20] Mackinnon MJ Read AF Virulence in malaria: an evolutionary viewpoint Philos Trans R Soc Lond B Biol Sci 2004 359 965 986 10.1098/rstb.2003.1414 15306410 PMC1693375

