# Supplementary figures and images for: Gestational malaria associated to Plasmodium vivax and Plasmodium falciparum placental mixed-infection followed by foetal loss: a case report from an unstable transmission area in Brazil
Source: Malar J. 2011 Jun 27;10:178. doi: 10.1186/1475-2875-10-178 (PMC3141593; doi:10.1186/1475-2875-10-178)

MW Po+ Pm+ Pv + Pf+ S C-

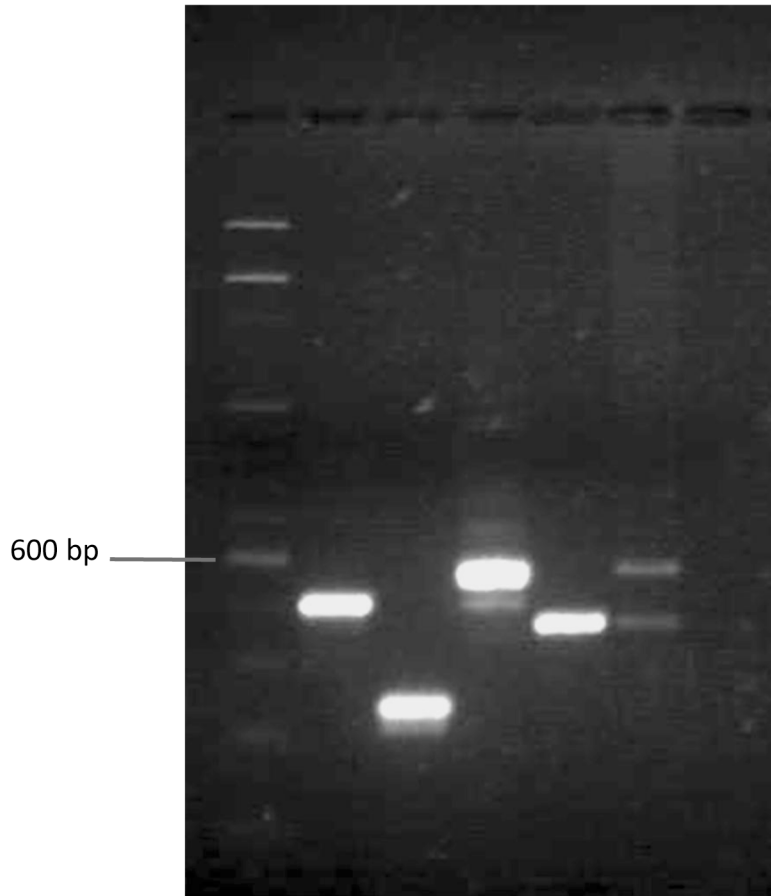

Supplement: Additional file 1 — Presence confirmation of P. falciparum- and P. vivax-infected erythrocytes in the placental tissue collected after foetal loss. Agarose gel electrophoresis of semi-nested multiplex PCR (SnM-PCR) amplified products in the presence of species-specific human malaria parasite oligonucleotides specific for P. ovale, P. malariae, P. vivax or P. falciparum ssrRNA gene. Both P. vivax and P. falciparum gDNA were found in the placenta. Abbreviations are as follows: MW, molecular weight; bp, base pair; S, patient placental sample; C-, negative control, absence of nuclear material; Po+, Pm+, Pv+ and Pf+, positive controls representing amplification product of P. ovale (436 bp), P. malariae (269 bp), P. vivax (499 bp) and P. falciparum (395 bp) ssrRNA gene. [file 1475-2875-10-178-S1.PDF]

VIV Oligonucleotides

FAL Oligonucleotides

**MW**

**B**

**niB**

**C-**

**B**

**niB**

**C-**

**Pv +**

**Pf +**

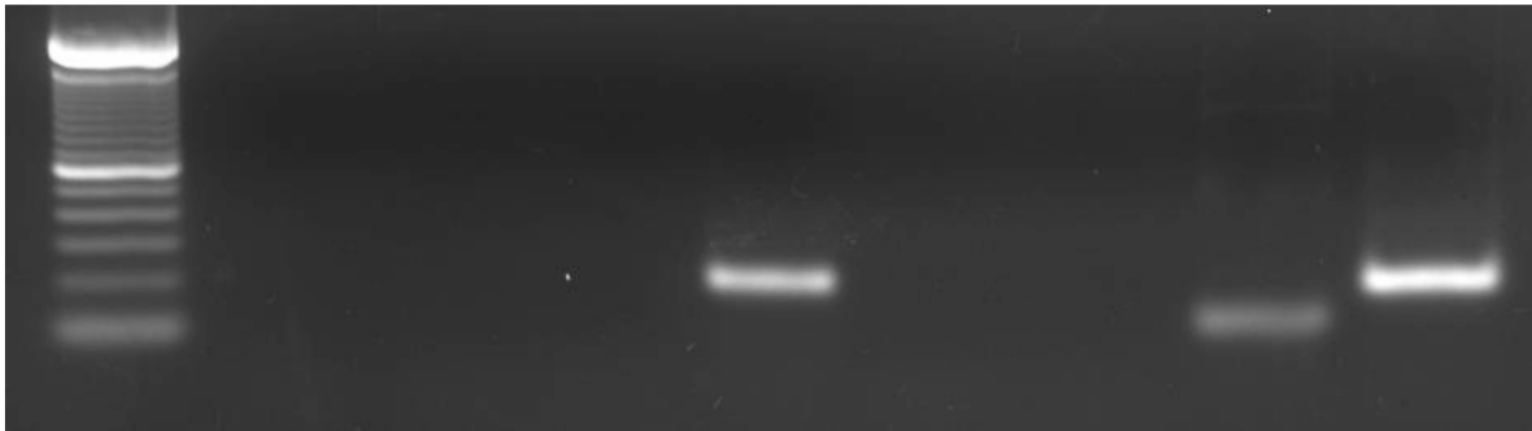

Supplement: Additional file 2 — Molecular diagnosis of Plasmodium falciparum infection performed two months after delivery. Agarose gel electrophoresis representing nested-PCR performed in the presence of patient's peripheral blood collected two months after delivery and species-specific human malaria parasite oligonucleotides (VIV or FAL, specific for P. vivax or P. falciparum ssrRNA gene, respectively). Only P. falciparum gDNA was detected in peripheral blood. Abbreviations are as follows: MW, molecular weight; bp, base pair; B, patient peripheral blood sample; niB, non-infected peripheral blood sample; C-, negative control, absence of nuclear material; Pv+ and Pf+, positive controls representing amplification product of P. vivax (120 bp) and P. falciparum (200 bp) ssrRNA gene. [file 1475-2875-10-178-S2.PDF]
